# The association among MDCT-derived three-dimensional visceral adiposities on cardiac diastology and dyssynchrony in asymptomatic population

**DOI:** 10.1186/s12872-015-0136-8

**Published:** 2015-10-30

**Authors:** Yau-Huei Lai, Charles Jia-Yin Hou, Chun-Ho Yun, Kuo-Tzu Sung, Cheng-Huang Su, Tung-Hsin Wu, Fei-Shih Yang, Ta-Chuan Hung, Chung-Lieh Hung, Hiram G. Bezerra, Hung-I Yeh

**Affiliations:** Division of Cardiology, Department of Internal Medicine, Mackay Memorial Hospital, No. 92, Sec. 2, Zhongshan N. Road, Taipei, Taiwan; Division of Cardiology, Department of Internal Medicine, Mackay Memorial Hospital, Hsinchu, Taiwan; Mackay Junior College of Medicine, Nursing, and Management, Taipei, Taiwan; Mackay Medical College, Taipei, Taiwan; Department of Radiology, Mackay Memorial Hospital, Taipei, Taiwan; Department of Biomedical Imaging and Radiological Sciences, National Yang Ming University, Taipei, Taiwan; The Institute of Health Policy and Management, College of Public Health, National Taiwan University, Taipei, Taiwan; University Hospitals Harrington Heart & Vascular Institute, Division of Cardiology, Case Western Reserve University, Cleveland, OH USA

**Keywords:** Pericardial fat, Diastolic dysfunction, Dyssynchrony, Tissue Doppler

## Abstract

**Background:**

Visceral adipose tissue, a biologically active fat depot, has been proposed as a reliable marker for visceral adiposity and metabolic abnormalities. Effects of such adiposity on LV diastolic function and dyssynchrony remained largely unknown.

**Methods:**

We assessed pericardial fat (PCF) and thoracic peri-aortic fat (TPAF) by three-dimensional (3D) volume-vender multi-detector computed tomography (MDCT) (Aquarius 3D Workstation, TeraRecon, San Mateo, CA, USA). Echo-derived diastolic parameters and tissue Doppler imaging (TDI) defined mitral annular systolic (S’), early diastolic (E’) velocities as well as LV filling (E/E’) were all obtained. Intra-ventricular systolic (Sys-D) and diastolic (Dias-D) dyssynchrony were assessed by TDI method.

**Results:**

A total of 318 asymptomatic subjects (mean age: 53.5 years, 36.8 % female) were eligible in this study. Greater PCF and TPAF were both associated with unfavorable diastolic indices and higher diastolic dyssynchrony (all *p* < 0.05). These associations remained relatively unchanged in multi-variate models. PCF and TPAF set at 81.68 & 8.11 ml yielded the largest sensitivity and specificity (78.6 and 60 % for PCF, 75 and 66.6 % for TPAF, respectively) in predicting abnormally high LV diastolic dyssynchrony, which was defined as Dias-D≧55 ms.

**Conclusion:**

Increasing visceral adiposity may be associated with adverse effects on myocardium, primarily featured by worse diastolic function and greater degree of dyssynchrony.

**Electronic supplementary material:**

The online version of this article (doi:10.1186/s12872-015-0136-8) contains supplementary material, which is available to authorized users.

## Background

Heart failure (HF) has emerged as a rapid growing epidemic in recent years [1]. There has been increasing academic interest in heart failure with preserved ejection fraction (HFpEF), which may account for nearly half of the HF population [2]. Recent studies have shown that mechanical dyssynchrony, an important pathophysiologic surrogate associated with myocardial asynergy, is not solely limited to systolic heart failure, but also exists in HFpEF and normal QRS duration [3, 4]. Diastolic dysfunction and its progression are independent predictors of incident heart failure [5].

Accumulating epidemiologic data have recently suggested that metabolic abnormalities accompanied by obesity, excessive body fat and systemic inflammation can confer higher heart failure risks [6–9], partially through left ventricular (LV) remodeling or hypertrophy [10, 11]. Obesity is associated with altered LV remodeling, possibly due to increased hemodynamic load, neurohormonal activation, and increased cytokine production [12]. A recent study showed that overweight and obesity were independent predictors of LV diastolic dysfunction [13]. However, the mechanistic link between excessive visceral adiposity and LV mechanical asynergy, especially diastolic dysfunction, remained largely unexplored.

Pericardial fat (PCF), which can be accurately quantified by various imaging tools and serve as a reliable marker of visceral adiposity [14, 15], has been recently proposed as an active source of proatherogenic cytokines that mediate systemic inflammation and metabolic derangements in obese subjects [16]. It had also been shown to influence LV structure and function through mechanical or paracrine effects, partially due to its anatomic proximity to the myocardium [17]. We sought to investigate the association between visceral adiposity and LV diastolic indices in subjects undergoing cardiovascular health survey.

## Methods

### Subjects

From 2009 to 2013, we consecutively studied subjects who attended primary cardiovascular health survey in a tertiary medical center in Taipei, Taiwan. Participants with decompensated heart failure, unstable coronary events, symptomatic angina or those who underwent hemodialysis were precluded from this primary screen program. Detailed physical examination was performed as well as a thorough review of baseline characteristics, medical history including current smoking behavior and regular physical activity from structured questionnaires. Subjects with known cardiovascular disease history defined as previous myocardial infarction, coronary arterial disease, stroke, prior hospitalization for congestive heart failure and peripheral arterial disease were excluded. Subjects with history of atrial fibrillation, left bundle branch block or pacemaker implantation were also excluded. A total of 318 subjects after exclusion during study period were finally enrolled.

Hypertension was defined as systolic blood pressure higher than 140 mmHg, diastolic blood pressure higher than 90 mmHg or previously diagnosed hypertension under medication control. Diabetes was defined as fasting glucose level more than 126 mg/dL or previously diagnosed diabetes under medication control. Hyperlipidemia was defined as those with known history and/or who use lipid-lowering drugs, such as statins or fibrates, on a daily basis.

This study has been approved by Mackay Memorial Hospital Institutional Review Board with adherence to research ethics (IRB number: 14MMHIS161). All participants were adults and provided written informed consent to be included in the study.

### Lab data acquisition and analysis

Hitachi 7170 Automatic Analyzer (Hitachi Corp. Hitachinaka Ibaraki, Japan) was used to measure fasting and postprandial glucose levels (hexokinase method), HbA1c, creatinine (kinetic colorimetric assay), total cholesterol and triglyceride (TG). Lipid profiles including low-density lipoprotein (LDL) and high-density lipoprotein cholesterol (HDL) were obtained by homogenous enzymatic colorimetric assay. High-sensitivity CRP (hs-CRP) levels were determined by using a highly sensitive, latex particle-enhanced immunoassay Elecsys 2010 (Roche, Mannheim, Germany).

### Echocardiographic assessment

Each subject underwent two-dimensional and color Doppler transthoracic echocardiogram (GE, Vivid 7, Vingmed Ultrasound, Norway) equipped with 2–4 MHz transducer at left decubitus position. Standardized echocardiography imaging protocol including measurement of left atrial dimension (LAD), wall thickness, LV volumes (biplane Simpson method), and LV mass (American Society of Echocardiography criteria) [18] were all obtained. LV diastolic function was determined by pulsed-wave Doppler of transmitral inflow early (E) and late diastolic (A) LV filling velocities measured at the tip of the mitral leaflets in the apical 4-chamber view. Diastolic functional parameters including early mitral inflow velocity (E), early-to-late inflow ratio (E/A), deceleration time (DT), isovolumetric relaxation time (IVRT), and spectral tissue Doppler imaging (TDI) defined mitral annulus systolic (S’) and early diastolic (E’) velocities were all obtained. The average values of S’ and E’ at LV basal lateral and septal segments were presented throughout this manuscript. Grading of diastolic dysfunction was then determined according to EAE/ASE recommendations [19].

For pulsed wave TDI-based measures, highest temporal resolution with frame rates >100 frames/sec with caution to optimize the parallel alignment for TDI tracing with myocardial longitudinal motion was achieved for raw imaging recording for subsequent dyssynchrony analysis. Intra-ventricular horizontal dyssynchrony was presented as the absolute time-to-peak velocity difference of S’ and E’ (Sys-D, Dias-D) between LV basal lateral and septal segments from apical 4-chamber views (Additional file 1: Figure S1). Significant diastolic dyssynchrony was defined as Dias-D≧55 ms based on a previous article [20].

### Measurement of pericardial fat

Multidetector computed tomography (MDCT) study was performed by a 16-slice scanner (Sensation 16, Siemens Medical Solutions, Forchheim, Germany) with 16 mm × 0.75 mm collimation, rotation time 420 ms and tube voltage of 120 kV. In one breath-hold, images were acquired from above the level of tracheal bifurcation to below the base of heart using prospectively ECG triggering with the centre of the acquisition at 70 % of the R-R interval. From the raw data, the images were reconstructed with standard kernel in 3 mm thick axial, non-overlapping slices and 25 cm field of view. Pericardial fat (PCF) and thoracic peri-aortic fat (TPAF) were quantified using a dedicated workstation (Aquarius 3D Workstation, TeraRecon, San Mateo, CA, USA). The semi-automated segmentation technique was developed for quantification of fat volumes. We traced the region of interest manually and defined fat tissue as pixels within a window of −195 to −45 HU and a window centre of −120 HU. PCF was defined as any adipose tissue located within the pericardium from the level of left main coronary artery to the cardiac base (Additional file 2: Figure S2A and 2B). TPAF was defined as all of the adipose tissue surrounding the descending thoracic aorta which extended 67.5 mm from the level of the bifurcation of pulmonary arteries with cranial-caudal coverage. This approach has previously been validated [21].

### Statistical analysis

Continuous data was expressed as the mean and standard deviation with categorical data expressed as the frequency and proportion of occurrence in all subjects. Differences of baseline demographics, metabolic and echocardiographic parameters between PCF and TPAF tertile groups were tested by ANOVA test with categorical data analyzed by chi-square or Fisher’s exact test as appropriate. Uni- and multivariable regression models including confounding variables of age, gender, BMI, blood pressure, LV mass index and clinical histories (hypertension, diabetes, hyperlipidemia, smoking habits) were used to determine the independent association between adipose tissue volumes (both PCF & TPAF), LV structural/functional parameters and degree of dyssynchrony (Sys-D/Dias-D). The area under the receiver-operator characteristic curve (AUROC) for PCF/TPAF in identifying significant LV septal and lateral wall (systolic and diastolic) timing differences was used as a summary measure of clinical outcome measure for dyssynchrony (defined as ≧60 & 55 ms for systolic and diastolic dyssynchrony, respectively), with corresponding 95 % confidence interval (CI) was further reported.

All data were analyzed using a commercial software STATA 8.2 package (Stata Corp., College Station, Texas). The significance of p level (α-value) for all analysis was two-sided, with a value less than 0.05 considered statistically significant.

## Results

### Baseline demographics and the association between visceral adiposity and clinical information

Of all 318 subjects (mean age: 53.5 years, 36.8 % female) enrolled, 102 (32.1 %) had hypertension, 46 (14.5 %) had diabetes, and 109 (34.3 %) had hyperlipidemia with a mean PCF and TPAF volume of 80.6 ± 33 & 7.89 ± 4.47 ml, respectively. Median PCF volume was 75.98 ml (interquartile range = 39.02), and median TPAF volume was 7.02 ml (interquartile range = 5.08). The association between baseline demographics, biochemical data and visceral adiposity are summarized in the first half of Table 1. In brief, both higher PCF and TPAF were associated with higher systolic blood pressure, male predominance and decreased HDL levels (all *p* < 0.05). There were also significant differences in age and diastolic blood pressure across PCF tertile groups (both *p* < 0.05).

**Table 1:** Baseline demographics, biomarkers and echocardiographic parameters categorized by pericardial and periaortic fat volumes

PCF, ml	Q1 (*n* = 106)	Q2 (*n* = 106)	Q3 (*n* = 106)	F	*p*-value
	<63.6 ml	63.6-88.7 ml	>88.7 ml		
Age (years)	51.67 ± 8.81	53.60 ± 9.98	55.40 ± 10.29	3.609	0.028
Male	56 (52.8 %)	72 (67.9 %)	73 (68.9 %)	-	0.025
BMI (kg/m^2^)	24.78 ± 3.50	25.48 ± 3.97	25.40 ± 3.41	0.787	0.456
SBP (mmHg)	119.04 ± 17.68	126.30 ± 16.88	127.15 ± 15.20	8.193	<0.001
DBP (mmHg)	74.42 ± 11.17	77.97 ± 9.99	77.62 ± 10.27	5.121	0.006
HbA1c (%)	5.97 ± 0.75	5.97 ± 0.62	6.08 ± 0.73	1.242	0.29
Cholesterol (mg/dL)	201.27 ± 37.52	203.14 ± 37.28	205.33 ± 42.73	0.977	0.378
TG (mg/dL)	137.70 ± 77.21	149.98 ± 90.00	144.72 ± 94.28	0.733	0.481
HDL (mg/dL)	51.50 ± 13.96	50.07 ± 14.13	48.72 ± 13.67	4.12	0.017
LDL (mg/dL)	130.75 ± 33.79	132.18 ± 33.79	135.29 ± 33.23	1.595	0.205
Hs-CRP (mg/dL)	0.174 ± 0.022	0.255 ± 0.035	0.26 ± 0.043	1.312	0.271
BNP (pg/mL)	33.62 ± 6.33	25.64 ± 4.23	32.88 ± 7.19	0.532	0.588
Hypertension	28 (26.4 %)	35 (33 %)	39 (36.8 %)	-	0.241
Diabetes	13 (12.3 %)	14 (13.2 %)	19 (17.9 %)	-	0.443
Hyperlipidemia	33 (31.1 %)	42 (39.6 %)	34 (32.1 %)	-	0.236
LAD (mm)	28.72 ± 5.62	30.51 ± 7.57	32.11 ± 5.15	7.956	<0.001
LVEF (%)	67.81 ± 4.27	67.61 ± 4.92	67.08 ± 5.2	0.657	0.519
LVEDV (ml)	101.03 ± 17.3	103.46 ± 18.97	105.718.91	1.708	0.183
LVESV (ml)	32.36 ± 6.35	33.75 ± 9.14	35.06 ± 9.78	2.622	0.074
LVMI (g/m^2^)	83.57 ± 14.77	81.03 ± 23.78	84.03 ± 22.36	0.585	0.055
IVS (mm)	9.89 ± 7.95	9.53 ± 1.44	9.88 ± 1.33	0.198	0.028
PWT (mm)	9.84 ± 7.93	9.26 ± 1.37	9.81 ± 1.11	0.522	0.059
E (cm/s)	69.27 ± 15.13	62.3 ± 17.88	61.69 ± 15.39	7.42	0.001
DT (ms)	196.88 ± 32.98	208.7 ± 43	214.72 ± 49.06	4.211	0.016
IVRT (ms)	87.14 ± 13.58	90.91 ± 14.85	92.44 ± 16.49	3.588	0.028
E/A	1.18 ± 0.37	1.04 ± 0.47	0.94 ± 0.24	7.927	<0.001
S’ (cm/s)	7.54 ± 1.71	7.24 ± 1.5	7.11 ± 1.43	2.183	0.114
E’ (cm/s)	8.77 ± 2.06	7.68 ± 2.17	6.75 ± 1.6	28.32	<0.001
E/E’	7.17 ± 2.21	7.9 ± 4.03	8.49 ± 3.19	5.885	0.003
Diastolic dysfunction^a^	52 (49.1 %)	77 (72.6 %)	89 (84 %)	-	<0.001
Dias-D (ms)	21.42 ± 15.27	26.42 ± 22.18	32.06 ± 25.39	2.258	0.001
Sys-D (ms)	28.21 ± 21.42	31.79 ± 27.6	33.18 ± 29.09	1.838	0.161
TPAF, ml	Q1 (*n* = 106)	Q2 (*n* = 106)	Q3 (*n* = 106)	F	*p*-value
	<5.71 ml	5.71-8.53 ml	>8.53 ml		
Age (years)	52.74 ± 9.37	53.53 ± 9.88	54.42 ± 10.17	0.749	0.474
Male	42 (39.6 %)	74 (69.8 %)	85 (80.2 %)	-	<0.001
BMI (kg/m^2^)	24.9 ± 3.72	25.42 ± 3.46	25.31 ± 3.74	0.41	0.385
SBP (mmHg)	119.87 ± 17.14	125.48 ± 17.4	127.27 ± 15.51	4.259	0.015
DBP (mmHg)	75.21 ± 11.07	78.9 ± 10.21	75.95 ± 10.13	1.876	0.155
HbA1c (%)	5.96 ± 0.78	5.98 ± 0.64	6.08 ± 0.70	1.141	0.321
Cholesterol (mg/dL)	201.56 ± 36.87	204.7 ± 39.09	203.56 ± 41.8	0.133	0.876
TG (mg/dL)	135.06 ± 77.37	146.32 ± 82.82	151.18 ± 100.5	0.843	0.431
HDL (mg/dL)	52.75 ± 14.88	48.85 ± 13.22	48.68 ± 13.33	3.072	0.048
LDL (mg/dL)	129.99 ± 33.17	135.68 ± 34.56	132.64 ± 32.96	0.368	0.68
Hs-CRP (mg/dL)	0.167 ± 0.022	0.216 ± 0.049	0.312 ± 0.047	1.761	0.174
BNP (pg/mL)	26.41 ± 3.93	26.84 ± 5.17	38.89 ± 8.18	1.381	0.253
Hypertension	25 (23.6 %)	39 (36.8 %)	38 (35.8 %)	-	0.064
Diabetes	11 (10.4 %)	17 (16 %)	18 (17 %)	-	0.321
Hyperlipidemia	36 (33.9 %)	35 (33 %)	38 (35.8 %)	-	0.908
LAD (mm)	28.65 ± 6.55	30.3 ± 6.54	32.39 ± 5.32	9.765	<0.001
LVEF (%)	68.23 ± 4.05	67.14 ± 5.09	67.13 ± 5.16	1.851	0.159
LVEDV (ml)	99.98 ± 17.88	105.13 ± 17.79	105.08 ± 19.34	2.755	0.065
LVESV (ml)	31.8 ± 7.5	34.63 ± 8.27	34.74 ± 9.64	4.059	0.018
LVMI (g/m^2^)	82.11 ± 17.22	85.86 ± 21.18	80.69 ± 22.74	1.663	0.191
IVS (mm)	9.04 ± 1.49	9.48 ± 1.01	10.79 ± 7.88	4.048	0.018
PWT (mm)	8.93 ± 1.11	9.4 ± 1.3	10.58 ± 7.87	3.576	0.029
E (cm/s)	68.78 ± 19.08	65.44 ± 15.23	59.13 ± 13.31	9.465	<0.001
DT (ms)	198 ± 39.47	204.14 ± 38.78	218.22 ± 47.3	4.204	0.016
IVRT (ms)	89.77 ± 14.71	89.82 ± 13.85	90.9 ± 16.84	0.199	0.697
E/A	1.18 ± 0.46	1.06 ± 0.36	0.92 ± 0.28	12.42	<0.001
S’ (cm/s)	7.21 ± 1.52	7.38 ± 1.59	7.31 ± 1.57	0.328	0.072
E’ (cm/s)	8.39 ± 2.34	7.92 ± 1.95	6.89 ± 1.78	15.01	<0.001
E/E’	7.89 ± 4.05	7.61 ± 2.49	8.06 ± 3.08	0.921	0.399
Diastolic dysfunction^a^	59 (55.7 %)	70 (66 %)	89 (84 %)	-	<0.001
Dias-D (ms)	23.68 ± 18.89	27.21 ± 18.75	32.99 ± 25.63	3.263	0.003
Sys-D (ms)	32.64 ± 25.35	28.49 ± 26.43	32.06 ± 27.01	1.947	0.165

Data are presented as mean ± standard deviation or number (percentage)

^a^Diastolic dysfunction was determined according to EAE/ASE recommendations [19]

*Abbreviations*: *PCF* pericardial fat, *TPAF* thoracic periaortic fat *BMI* body-mass-index, *SBP* systolic blood pressure, *DBP* diastolic blood pressure, *TG* triglyceride, *LDL* low-density lipoprotein, *HDL* high-density lipoprotein, *Hs-CRP* high-senstivity C-reactive protein, *BNP* brain natriuretic peptide, *LVEF* left ventricular ejection fraction, *LVEDV* left ventricular end-diastolic volume, *LVESV* left ventricular end-systolic volume, *LAD* left atrial dimension (M-mode), *LVMI* left ventricular mass index, *IVS* interventricular septum thickness (M-mode), *PWT* posterior wall thickness (M-mode), *E* early mitral inflow velocity, *A* late mitral inflow velocity, *DT* deceleration time, *IVRT* isovolumetric relaxation time, *S*’ systolic tissue Doppler velocity, *E*’ early-diastolic tissue Doppler velocity, *Dias-D* E’ time-to-peak difference (diastolic dyssynchrony), *Sys-D* S’ time-to-peak difference (systolic dyssynchrony)

### The association between visceral adiposity, cardiac geometry and diastolic function

There were 148 subjects with Grade 1 diastolic dysfunction and 70 subjects with Grade 2 diastolic dysfunction. The association between echocardiographic data and visceral adiposity was summarized in the latter half of Table 1. Across tertile groups, greater PCF and TPAF were both associated with increased LAD, reduced E and E’ velocities, lower E/A ratio and prolonged DT (all *p* < 0.05). PCF was also associated with elevated E/E’ ratio, prolonged IVRT and increased interventricular septum thickness (all *p* < 0.05). TPAF was associated with significant increases in LV end-systolic volume and both wall thickness (all *p* < 0.05). There was no significant difference in LV ejection fraction (LVEF) with either adipose tissue. In Table 2, we re-categorized all subjects into with/without diastolic dysfunction groups according to EAE/ASE recommendations [19] and compared their key characteristics. There were significant differences in age, gender, both adipose tissue volumes, LA diameter and all Doppler parameters of diastolic function (all *p* < 0.05). Increased BNP was also borderlinely associated with diastolic dysfunction (*p* = 0.06).

**Table 2:** Baseline demographics, biomarkers and echocardiographic parameters of patients with and without diastolic dysfunction^a^

	Normal (*n* = 100)	Diastolic dysfunction (*n* = 218)	F	*p*-value
Age (years)	50.82 ± 9.34	54.83 ± 9.8	11.85	0.001
Male	60 (60 %)	141 (64.7 %)	-	0.453
PCF (ml)	67.32 ± 31.95	86.79 ± 31.77	25.68	<0.001
TPAF (ml)	6.32 ± 3.73	8.63 ± 4.61	19.24	<0.001
BMI (kg/m^2^)	25.05 ± 3.78	25.36 ± 4.14	0.415	0.52
HbA1c (%)	4.84 ± 2.44	4.84 ± 2.48	<0.001	0.992
Cholesterol (mg/dL)	195.77 ± 45.85	203.94 ± 42.61	2.399	0.122
LDL (mg/dL)	124.72 ± 42.02	124.98 ± 46.85	0.002	0.963
Hs-CRP (mg/dL)	0.116 ± 0.039	0.166 ± 0.034	1.323	0.251
BNP (pg/mL)	21.03 ± 4.91	35.16 ± 6.7	3.572	0.06
Hypertension	29 (29 %)	73 (33.5 %)	-	0.441
Diabetes	12 (12 %)	34 (15.6 %)	-	0.493
Hyperlipidemia	40 (40 %)	69 (31.7 %)	-	0.162
LAD (mm)	28.76 ± 6.59	31.22 ± 6.08	10.64	0.001
E (cm/s)	68.81 ± 15.22	62.24 ± 16.23	11.7	0.001
DT (ms)	199.95 ± 35.88	210.04 ± 45.27	3.85	0.045
IVRT (ms)	85.66 ± 13.66	92.41 ± 15.49	13.98	<0.001
E’ (cm/s)	10 ± 1.59	6.7 ± 1.42	344.91	<0.001
E/E’	7.04 ± 1.85	9.63 ± 3.09	60.51	<0.001
Dias-D (ms)	22.62 ± 18.2	30.42 ± 21.66	9.793	0.002

Data are presented as mean ± standard deviation or number (percentage)

^a^Diastolic dysfunction was determined according to EAE/ASE recommendations [19]

Abbreviations same as Table 1

**Fig. 1:**
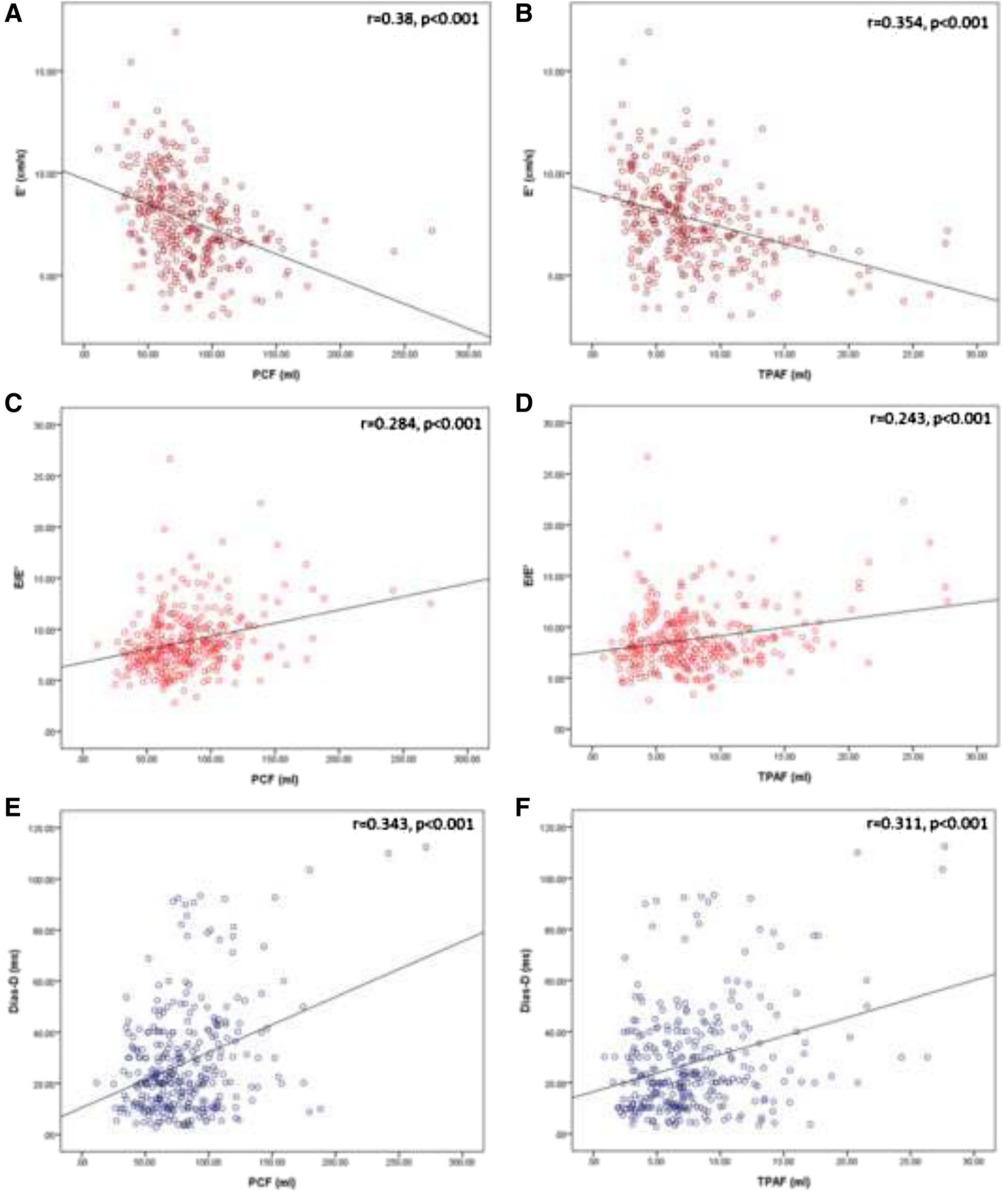
Linear regression scatter plots between E’ and PCF (**a**), TPAF (**b**), E/E’ and PCF (**c**), TPAF (**d**), Dias-D and PCF (**e**), TPAF (**f**). All plots showed excellent statistical significance (*p* < 0.001). Abbreviations: PCF = pericardial fat, TPAF = thoracic periaortic fat, E = early mitral inflow velocity, E’ = early-diastolic tissue Doppler velocity, Dias-D = E’ time-to-peak difference (diastolic dyssynchrony)

**Fig. 2:**
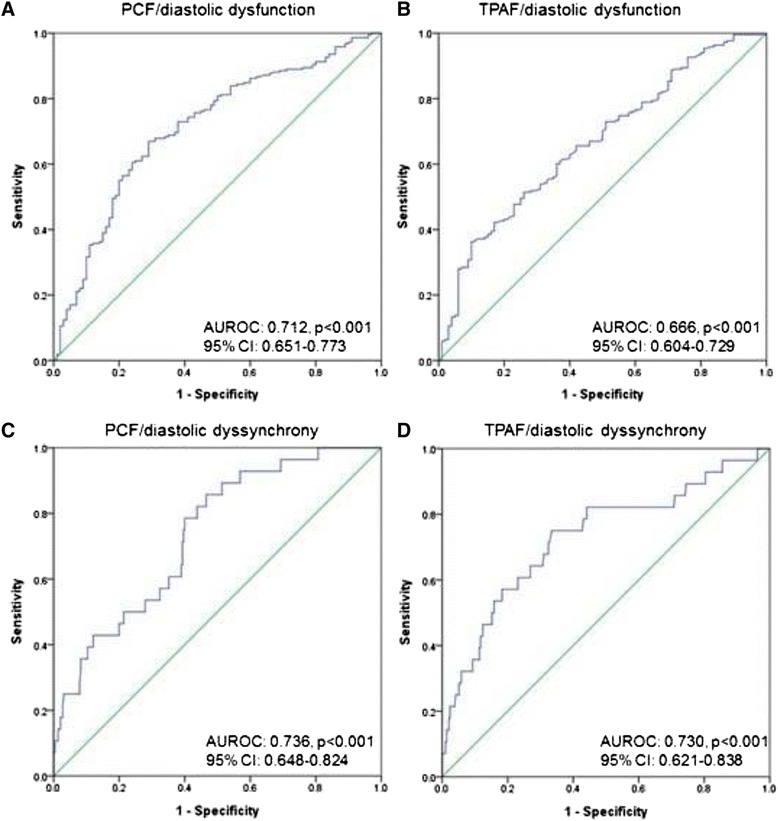
Receiver-operating characteristic curves. **a** PCF had optimal area under curve (AUROC) for identifying diastolic dysfunction (0.712, *p* < 0.001). **b** TPAF had modest AUROC for identifying diastolic dysfunction (0.666, *p* < 0.001). **c** PCF had optimal AUROC for identifying diastolic dyssynchrony (0.736, p < 0.001) defined as Dias-D≧55 ms. **d** TPAF had optimal AUROC for identifying diastolic dyssynchrony (0.730, *p* < 0.001)

Figure 1a-d demonstrated the linear regression plots of PCF/TPAF with E’ and E/E’. Both adipose tissue volumes had excellent correlation with these two key parameters of diastolic dysfunction (decreased E’, increased E/E’; all *p* < 0.001). In Fig. 2a and b, receiver-operating characteristic (ROC) analysis showed that both PCF and TPAF had modest area under curve (AUROC) for identifying diastolic dysfunction (0.712 & 0.666, 95 % CI: 0.651–0.773 & 0.604–0.729 for PCF & TPAF, respectively, both *p* < 0.001). PCF and TPAF set at 67.31 & 6.69 ml yielded the largest sensitivity and specificity (72.9 and 62 % for PCF, 62.8 and 60 % for TPAF, respectively) in predicting diastolic dysfunction. It should be noted here that the p values yielded by ANOVA (Table 1) and linear regression analysis (Fig. 1d) between TPAF and E/E’ seemed contradictory. This discrepancy might be attributed to non-normal distribution of TPAF.

In Table 3, we showed the uni- and multi-variate regression models regarding the association between visceral adiposity and various echocardiographic parameters. After control of multivariables including age, gender, BMI, systolic blood pressure, LV mass index and clinical histories (hypertension, diabetes, hyperlipidemia, smoking habits), both greater PCF and TPAF remained significantly associated with increased LAD, lower E/A ratio, reduced E’ velocity and elevated E/E’ ratio (all *p* < 0.01).

**Table 3:** Univariate and multivariate regression analyses of pericardial and periaortic fat volumes associated with echocardiographic parameters

PCF	Univariate model			Multivariate model^a^		
	B Coefficient (unstandardized)	95 % CI	*p*-value	B Coefficient (unstandardized)	95 % CI	*p*-value
LVEF	−0.008	−0.025-0.009	0.376	−0.003	−0.02-0.013	0.699
LAD	0.043	0.021-0.065	<0.001	0.04	0.018-0.063	<0.001
E	−0.015	−0.07-0.04	0.035	−0.021	−0.077-0.036	0.047
DT	0.197	0.049-0.346	0.009	0.148	−0.002-0.298	0.052
IVRT	0.067	0.015-0.12	0.013	0.063	0.008-0.117	0.025
E/A	−0.002	−0.003- -0.001	0.003	−0.002	−0.003- -0.001	0.006
E’	−0.024	−0.03- -0.017	<0.001	−0.02	−0.027- -0.013	<0.001
S’	−0.006	−0.012- -0.001	0.021	−0.005	−0.01- 0.001	0.084
E/E’	0.026	0.016-0.036	<0.001	0.02	0.011-0.03	<0.001
Dias-D	0.211	0.142-0.279	<0.001	0.197	0.128-0.266	<0.001
Sys-D	0.086	0.001-0.174	0.054	0.076	−0.014-0.166	0.096
TPAF	Univariate model			Multivariate model^a^		
	B Coefficient (unstandardized)	95 % CI	*p*-value	B Coefficient (unstandardized)	95 % CI	*p*-value
LVEF	−0.045	−0.172-0.082	0.487	−0.007	−0.131-0.117	0.914
LAD	0.377	0.215-0.539	<0.001	0.368	0.204-0.532	<0.001
E	−0.283	−0.692-0.127	0.175	−0.335	−0.754-0.085	0.118
DT	2.068	0.973-3.163	<0.001	1.712	0.606-2.819	0.003
IVRT	0.396	0.001-0.791	0.05	0.351	−0.056-0.758	0.091
E/A	−0.017	−0.026- -0.009	<0.001	−0.017	−0.026- -0.008	<0.001
E’	−0.164	−0.216- -0.111	<0.001	−0.137	−0.188- -0.086	<0.001
S’	−0.04	−0.081-0.001	0.05	−0.028	−0.069-0.012	0.173
E/E’	0.176	0.104-0.249	<0.001	0.134	0.064-0.203	<0.001
Dias-D	1.461	0.947-1.976	<0.001	1.367	0.848-1.885	<0.001
Sys-D	0.489	−0.165-1.143	0.142	0.415	−0.258-1.088	0.225

^a^Adjusted for age, gender, BMI, systolic blood pressure, LV mass index and clinical histories (hypertension, diabetes, hyperlipidemia, smoking habits)

Abbreviations same as Table 1

### The association between visceral adiposity and dyssynchrony

There were 28 subjects with abnormally high diastolic dyssynchrony (Dias-D≧55 ms) and 22 subjects with systolic dyssynchrony (Sys-D≧60 ms). As seen in Table 1, both higher PCF and TPAF were positively associated with higher degree of diastolic dyssynchrony across tertile groups (both *p* < 0.01). In Fig. 1e and f, linear regression plots also showed excellent correlation between both adipose tissue volumes and diastolic dyssynchrony (both *p* < 0.001). In the uni- and multi-variate regression models of Table 3, we consistently demonstrated the association between both visceral adiposities and diastolic dyssynchrony (adjusted B coefficient: 0.197 & 1.367 for PCF & TPAF, respectively, both *p* < 0.001).

Finally in ROC analysis, both PCF and TPAF had optimal AUROC for identifying diastolic dyssynchrony (0.736 & 0.730, 95 % CI: 0.648–0.824 & 0.621–0.838 for PCF & TPAF, respectively, both *p* < 0.001), when defined by the aforementioned criteria (Dias-D≧55 ms). PCF and TPAF set at 81.68 & 8.11 ml yielded the largest sensitivity and specificity (78.6 and 60 % for PCF, 75 and 66.6 % for TPAF, respectively) in predicting abnormally high LV diastolic dyssynchrony (Figure 2c and d).

## Discussion

In the present study, we observed that both pericardial and peri-aortic fat accumulations were significantly associated with diastolic dysfunction and dyssynchrony independent of LV mass and traditional risk factors. This association was slightly tighter with pericardial fat. We also identified their respective cutoff values for predicting diastolic dysfunction and dyssynchrony. To our knowledge, this is the first study to explore the relationship between such visceral adiposities and dyssynchrony by tissue Doppler imaging.

Our team has reported previously that pericardial fat was independently associated with metabolic derangements, fatty liver disease and systemic inflammation [14]. Although in this study, the association between pericardial fat and baseline metabolic risk factors was mostly weak. This may be explained by the relatively small sample size and benign clinical status of our participants. There was also a lack of correlation with BMI. Another bivariate analysis between BMI and E/E’ showed no significant correlation either (*r* = 0.045, *p* = 0.423), which is contrary to most worldwide epidemiologic studies. One possible explanation of our unusual findings might be that our average BMI is relatively normal (only 28 subjects had BMI > 30). On the other hand, the strong correlation that we demonstrated with advanced tissue Doppler metrics suggested a specific process linking pericardial fat to subclinical diastolic dysfunction that might be initiated much earlier than systemic metabolic derangements or excessive body mass accumulation. The specific effects of pericardial fat on cardiac structure and function via various mechanisms have been proposed in previous literature [22]. Compression of the heart by this enveloping fat deposit may also cause impaired LV diastolic filling, leading to atrial remodeling and dilation [17]. Pericardial fat also contains high levels of pro-atherogenic cytokines [23] that may induce inflammation and collagen turnover, leading to ventricular stiffness and diastolic dysfunction [24, 25].

Besides LA enlargement, we also observed a slight but significant increase in LV wall thickness among subjects with larger PCF or TPAF. Although diastolic dysfunction or dyssynchrony is not uncommon in people with normal wall thickness, LV hypertrophy and hypertensive heart disease are indeed the most important causes of HFpEF [26, 27]. Therefore, it is reasonable that these changes can coexist with visceral fat accumulation and diastolic dysfunction or dyssynchrony. On the other hand, no significant difference was found with LV mass index, and multivariate regression analysis also showed that LV mass index was a nonfactor, indicating that our primary findings were independent of LV hypertrophy.

Recently, it has been discovered that dyssynchrony is not as uncommon as previously assumed, and can occur to some extent even in the normal heart [28]. Due to its excellent temporal resolution, TDI has been widely utilized for quantifying the degree of intra-ventricular mechanical dyssynchrony. Through assessment of systolic myocardial velocity, TDI has also demonstrated the presence of subclinical systolic dysfunction in HFpEF [29], hypertensive heart disease and left ventricular hypertrophy [30].

As previously mentioned, both systolic and diastolic dyssynchrony have been found to exist in HFpEF, though diastolic dyssynchrony does seem to be more prevalent and may occur exclusively [3, 4]. This is evident with the sole presence of diastolic dyssynchrony in our study population. Several possible mechanisms have been suggested. First, early diastolic filling is mainly determined by the coordination of its previous systolic phase. Discordance of systolic contraction will lead to prolonged relaxation and reduced diastolic filling time [31]. Second, increased afterload may contribute to diastolic dyssynchrony through elevated wall stress and myocardial oxygen demand, leading to uneven distributions in coronary blood flow [32, 33]. Dyssynchrony has been reported to be associated with LV filling pressure in asymptomatic hypertensive patients with normal QRS duration and EF [34]. Other etiologies include conduction system disease, hypertrophy or fibrosis.

One recent study using strain imaging showed that obesity is a significant independent predictor of intra-ventricular dyssynchrony [35]. Another TDI-related study showed significant reductions in myocardial velocities, global and regional strain among obese subjects [36]. There has been growing interest in the potentially toxic effects of “cardiac steatosis” (excessive deposition of triglycerides in the myocardial cells) [37], which may induce myocardial fibrosis, cellular apoptosis and mitochondrial dysfunction via free fatty acid turnover process [38, 39]. Cardiac steatosis may also be an independent predictor of diastolic dysfunction in diabetic patients [40].

Due to its proximity to the myocardium and the same coronary blood supply, pericardial fat may have stronger correlations with coronary vasculopathy [41], cardiac structure and function than other measures of adiposity, such as total visceral fat or subcutaneous fat tissue [42]. The current study results suggest that visceral fat adjacent to cardiac structures may have an independent role in mediating regional disturbances of coronary supply and cardiac steatosis, resulting in the development of intra-ventricular dyssynchrony. Additionally, the independent association between pericardial fat burden and increased LA dimension, an important clinical feature and predictor of HF mechanics beyond ventricular hypertrophy [43], further highlighted the possible role of regional adipose depots in mediating HF development.

### Limitations

There are several limitations in our study. The first, our study has a male gender predominance, which may be somewhat biased. Secondly, this survey is retrospective and cross-sectional, without longitudinal follow up or validation with clinical outcomes. Thirdly, our data come from asymptomatic Asian participants who underwent primary cardiovascular health survey, and who therefore may not be fully representative of the broader general population in daily out-patient clinics. Finally, only basal septal and lateral segments were used to calculate dyssynchrony, which may be an overly simplistic estimate. We also acknowledge that our data regarding LA remodeling were mainly focused on LA diameter rather than 2-dimensional echo-based volume measurements.

## Conclusion

Our study provides valuable insights into the underlying of potential pathophysiologic mechanisms that visceral adipose tissue may exert several biological effects on myocardial contractile or diastolic functions and coordination, leading to impaired diastolic dysfunction, more elevated filling pressures and prolonged dyssynchrony, even in asymptomatic subjects. Our findings are critical in the conceptual framework of current understanding in obesity or metabolic derangements related myocardial asynergy and predisposition to preserved ejection fraction HF development. Excessive visceral adiposity burden linked to metabolic derangements, when assessed by CT, may thus serve as a possible marker or target for associated diagnostic purposes and future therapeutic intervention in these population.

## Additional files

Additional file 1: Figure S1. Pulsed-wave tissue Doppler measurement of intra-ventricular dyssynchrony. Upper panel: lateral wall TDI waveform. Lower panel: medial wall TDI waveform. The time intervals between QRS onset and peak of S’/E’ were measured respectively. Systolic dyssynchrony was presented as the absolute time-to-peak difference of S’ between lateral and medial segments (T1-T3). Diastolic dyssynchrony was presented as the absolute time-to-peak difference of E’ (T2-T4). Abbreviations: Sl = lateral systolic myocardial velocity, El = lateral early-diastolic myocardial velocity, Sm = medial systolic myocardial velocity, Em = medial early-diastolic myocardial velocity. (TIFF 799 kb) Additional file 2: Figure S2. 3D-reconstruction of total pericardial fat volume from axial, sagittal and coronal images. Pericardial fat (yellow color) was selected as all adipose tissue within the pericardial sac and subtracted from the adjacent cardiac structures. (A) A case with large pericardial fat (total volume = 174.3 ml). This subject has decreased diastolic tissue velocity (lateral E’ = 4 cm/s) and prolonged diastolic dyssynchrony (80 ms). (B) A case with small pericardial fat (total volume = 26.39 ml). This subject has normal diastolic tissue velocity (lateral E’ = 12 cm/s) and diastolic dyssynchrony (10 ms). (ZIP 1204 kb)

## Abbreviations

HF: Heart failure
HFpEF: Heart failure with preserved ejection fraction
LV: Left ventricular
PCF: Pericardial fat
E: Early mitral inflow velocity
A: Late mitral inflow velocity
DT: Deceleration time
IVRT: Isovolumetric relaxation time
TDI: Tissue Doppler imaging
S’: Systolic tissue Doppler velocity
E’: Early-diastolic tissue Doppler velocity
Sys-D: S’ time-to-peak difference (systolic dyssynchrony)
Dias-D: E’ time-to-peak difference (diastolic dyssynchrony)
MDCT: Multidetector computed tomography
TPAF: Thoracic periaortic fat
