# Supplementary figures and images for: The association among MDCT-derived three-dimensional visceral adiposities on cardiac diastology and dyssynchrony in asymptomatic population
Source: BMC Cardiovasc Disord. 2015 Oct 30;15:142. doi: 10.1186/s12872-015-0136-8 (PMC4628304; doi:10.1186/s12872-015-0136-8)

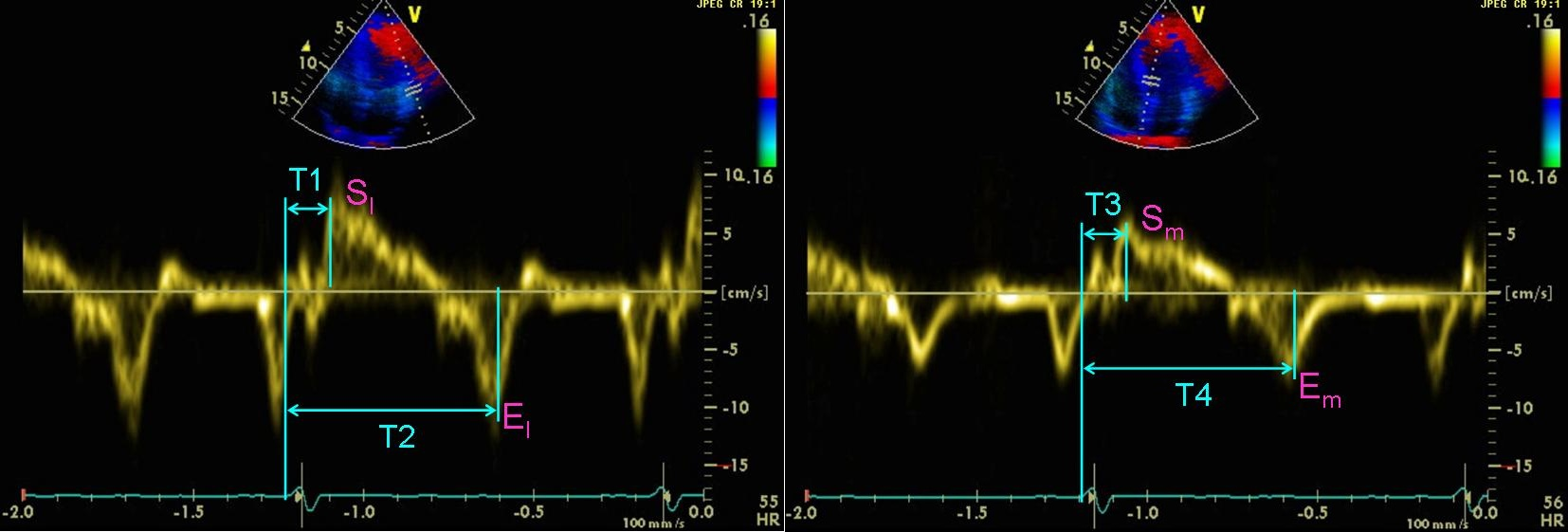

Supplement: Additional file 1: Figure S1. — Pulsed-wave tissue Doppler measurement of intra-ventricular dyssynchrony. Upper panel: lateral wall TDI waveform. Lower panel: medial wall TDI waveform. The time intervals between QRS onset and peak of S’/E’ were measured respectively. Systolic dyssynchrony was presented as the absolute time-to-peak difference of S’ between lateral and medial segments (T1-T3). Diastolic dyssynchrony was presented as the absolute time-to-peak difference of E’ (T2-T4). Abbreviations: Sl = lateral systolic myocardial velocity, El = lateral early-diastolic myocardial velocity, Sm = medial systolic myocardial velocity, Em = medial early-diastolic myocardial velocity. (TIFF 799 kb) [file 12872_2015_136_MOESM1_ESM.tif]

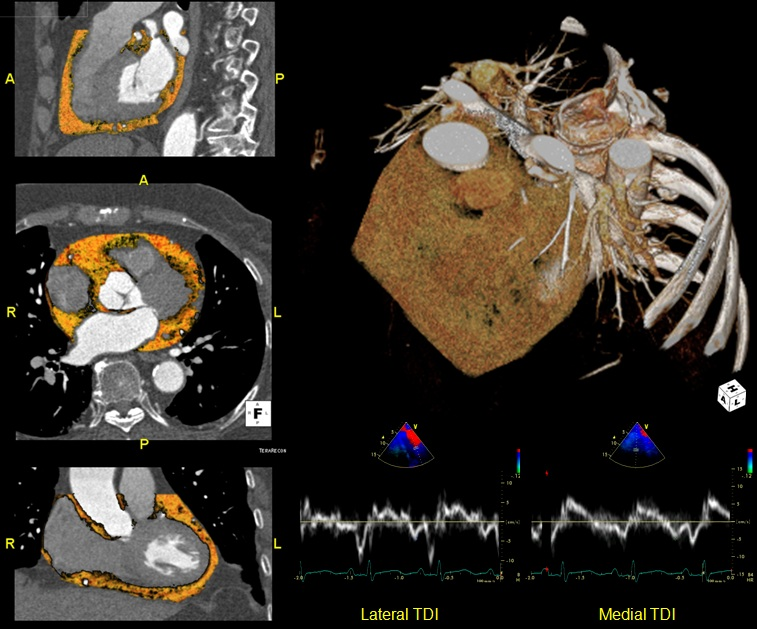

Supplement: Additional file 2: Figure S2. — 3D-reconstruction of total pericardial fat volume from axial, sagittal and coronal images. Pericardial fat (yellow color) was selected as all adipose tissue within the pericardial sac and subtracted from the adjacent cardiac structures. (A) A case with large pericardial fat (total volume = 174.3 ml). This subject has decreased diastolic tissue velocity (lateral E’ = 4 cm/s) and prolonged diastolic dyssynchrony (80 ms). (B) A case with small pericardial fat (total volume = 26.39 ml). This subject has normal diastolic tissue velocity (lateral E’ = 12 cm/s) and diastolic dyssynchrony (10 ms). (ZIP 1204 kb) [file 12872_2015_136_MOESM2_ESM.zip › Supplementary Figure 2AR3.tif]

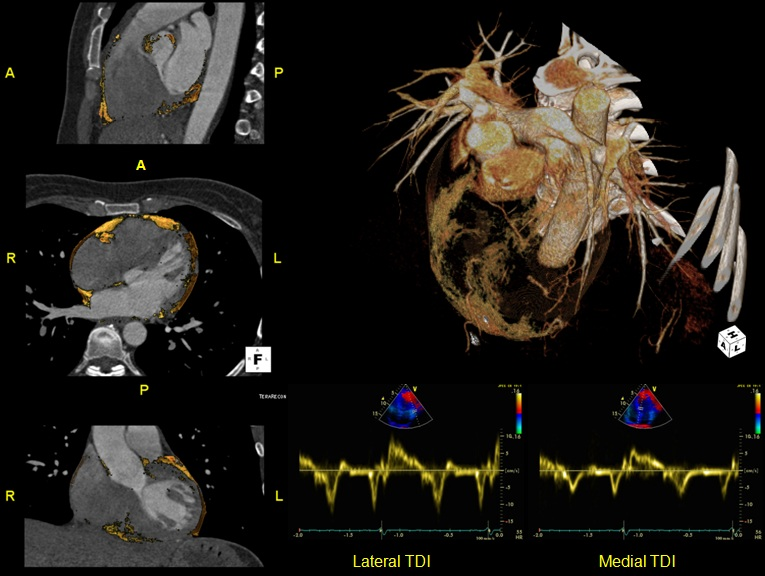

Supplement: Additional file 2: Figure S2. — 3D-reconstruction of total pericardial fat volume from axial, sagittal and coronal images. Pericardial fat (yellow color) was selected as all adipose tissue within the pericardial sac and subtracted from the adjacent cardiac structures. (A) A case with large pericardial fat (total volume = 174.3 ml). This subject has decreased diastolic tissue velocity (lateral E’ = 4 cm/s) and prolonged diastolic dyssynchrony (80 ms). (B) A case with small pericardial fat (total volume = 26.39 ml). This subject has normal diastolic tissue velocity (lateral E’ = 12 cm/s) and diastolic dyssynchrony (10 ms). (ZIP 1204 kb) [file 12872_2015_136_MOESM2_ESM.zip › Supplementary Figure 2BR3.tif]
